# Supplementary material for: Tucuxi-BLAST: Enabling fast and accurate record linkage of large-scale health-related administrative databases through a DNA-encoded approach
Source: PeerJ. 2022 Jul 11;10:e13507. doi: 10.7717/peerj.13507 (PMC9281601; doi:10.7717/peerj.13507)
Supplement: Supplemental Information 1 — The performance metrics for each program used for the Record Linkage in gold standard data [file peerj-10-13507-s001.docx]

**Supplementary Table 1: Benchmark (related to Figure 4)**

Contains the performance metrics for each program used for the Record Linkage in gold standard data

**Columns Description**

method Program(Method) used for Record Linkage

database gold standard data for HIV, meningitis (MEN) and tuberculosis (TB)

accuracy mean accuracy

sd_accuracy standard deviation for the accuracy

time_segs mean time in seconds

sd_time_segs standard deviation for the time in seconds

mem_gb mean memory comsuption in gigabytes

sd_mem_gb standard deviation for the the memory comsuption

sensitivity mean sensitivity

sd_sensitivity standard deviation for the sensitivity

especificity mean specificity

sd_especificity standard deviation for the specificity

f1-score mean f1-score

sd_f1-score standard deviation for the f1-score

| **method** | **database** | **accuracy** | **sd_accuracy** | **time_segs** | **sd_time_segs** | **mem_gb** | **sd_mem_gb** | **sensitivity** | **sd_sensitivity** | **especificity** | **sd_especificity** | **f1-score** | **sd_f1-score** |
| --- | --- | --- | --- | --- | --- | --- | --- | --- | --- | --- | --- | --- | --- |
| fastLink (blocking) | HIV | 83,97 | 0 | 5,57 | 0,03 | 1,37 | 0 | 81,84 | 0,00 | 100,00 | 0,00 | 90,01 | 0,03 |
| fastLink (blocking) | MEN | 74,38 | 0 | 3,32 | 0,06 | 1,39 | 0,49 | 73,60 | 0,00 | 100,00 | 0,00 | 84,80 | 0,06 |
| fastLink (blocking) | TB | 81,9 | 0 | 6,98 | 1,16 | 1,43 | 0,07 | 80,60 | 0,00 | 100,00 | 0,00 | 89,26 | 1,16 |
| fastLink (full) | HIV | 99,15 | 0 | 469,31 | 5,92 | 10,61 | 0,15 | 99,03 | 0,00 | 100,00 | 0,00 | 99,51 | 0,00 |
| fastLink (full) | MEN | 98,52 | 0 | 90,69 | 4,12 | 3,96 | 0 | 98,48 | 0,00 | 100,00 | 0,00 | 99,23 | 0,00 |
| fastLink (full) | TB | 98,63 | 0 | 595,87 | 6,93 | 13,5 | 0,27 | 98,53 | 0,00 | 100,00 | 0,00 | 99,26 | 0,00 |
| RecordLinkage R (blocking) | HIV | 77,03 | 0 | 2,63 | 0,03 | 0,28 | 0 | 74,09 | 0,00 | 99,84 | 0,00 | 85,06 | 0,00 |
| RecordLinkage R (blocking) | MEN | 62,56 | 0 | 2,6 | 0,01 | 0,27 | 0 | 61,42 | 0,00 | 100,00 | 0,00 | 76,10 | 0,00 |
| RecordLinkage R (blocking) | TB | 73,77 | 0 | 2,66 | 0,03 | 0,28 | 0 | 71,98 | 0,00 | 99,88 | 0,00 | 83,67 | 0,00 |
| RL Python (blocking) | HIV | 85,04 | 0 | 4,07 | 0,03 | 1,61 | 0,01 | 83,05 | 0,00 | 100,00 | 0,00 | 90,74 | 0,00 |
| RL Python (blocking) | MEN | 74,38 | 0 | 4,04 | 0,06 | 1,59 | 0,01 | 73,60 | 0,00 | 100,00 | 0,00 | 84,80 | 0,00 |
| RL Python (blocking) | TB | 82,62 | 0 | 4,1 | 0,04 | 1,81 | 0,07 | 81,38 | 0,00 | 100,00 | 0,00 | 89,73 | 0,00 |
| RL Python (full) | HIV | 88,89 | 0 | 260,1 | 12,19 | 21,02 | 0,06 | 87,41 | 0,00 | 100,00 | 0,00 | 93,28 | 0,00 |
| RL Python (full) | MEN | 80,3 | 0 | 54,05 | 4,42 | 6,22 | 0,03 | 79,70 | 0,00 | 100,00 | 0,00 | 88,70 | 0,00 |
| RL Python (full) | TB | NA | NA | NA | NA | NA | NA | NA | NA | NA | NA | NA | NA |
| Dedupe | HIV | 90,05 | 3,1 | 244,9 | 8,96 | 1,87 | 0,26 | 88,80 | 3,51 | 99,92 | 0,10 | 94,00 | 1,99 |
| Dedupe | MEN | 90,74 | 2,76 | 81 | 18,82 | 0,92 | 0,1 | 90,46 | 2,84 | 100,00 | 0,00 | 94,97 | 1,58 |
| Dedupe | TB | 91,04 | 2,76 | 308,6 | 18,51 | 2,43 | 0,16 | 90,48 | 4,47 | 99,90 | 0,10 | 94,91 | 2,53 |
| CIDACS-RL | HIV | 97,44 | 0 | 12,04 | 0,25 | 1,6 | 0,17 | 97,34 | 0,00 | 99,75 | 0,00 | 98,53 | 0,00 |
| CIDACS-RL | MEN | 92,61 | 0 | 8,73 | 0,15 | 1,41 | 0,16 | 92,89 | 0,00 | 99,46 | 0,00 | 96,06 | 0,00 |
| CIDACS-RL | TB | 95,82 | 0 | 12,9 | 0,13 | 1,65 | 0,26 | 95,86 | 0,00 | 99,64 | 0,00 | 97,72 | 0,00 |
| Tucuxi-BLAST (RF) | HIV | 98,47 | 0,05 | 4,44 | 0,08 | 0,43 | 0,06 | 99,38 | 0,07 | 98,89 | 0,05 | 99,14 | 0,03 |
| Tucuxi-BLAST (RF) | MEN | 98,52 | 0 | 2,21 | 0,06 | 0,38 | 0,07 | 98,98 | 0,00 | 99,49 | 0,00 | 99,23 | 0,00 |
| Tucuxi-BLAST (RF) | TB | 98,59 | 0,04 | 4,26 | 0,04 | 0,55 | 0,03 | 99,70 | 0,04 | 98,80 | 0,00 | 99,25 | 0,02 |
| Tucuxi-BLAST (LR) | HIV | 98,13 | 0,12 | 4,32 | 0,1 | 0,48 | 0,03 | 99,26 | 0,10 | 98,63 | 0,06 | 98,94 | 0,07 |
| Tucuxi-BLAST (LR) | MEN | 98,52 | 0 | 1,95 | 0,16 | 0,37 | 0,06 | 98,47 | 0,00 | 100,00 | 0,00 | 99,23 | 0,00 |
| Tucuxi-BLAST (LR) | TB | 98,62 | 0,06 | 4,14 | 0,07 | 0,55 | 0,03 | 99,39 | 0,07 | 99,13 | 0,07 | 99,26 | 0,03 |
